# Analyzing the causal relationship between lipid-lowering drug target genes and epilepsy: a Mendelian randomization study

**DOI:** 10.3389/fneur.2024.1331537

**Published:** 2024-03-08

**Authors:** Shicun Huang, Yuan Liu, Yi Zhang, Yiqing Wang, Ya Gao, Runnan Li, Lidong Yu, Xiaowei Hu, Qi Fang

**Affiliations:** ^1^Department of Neurology, First Affiliated Hospital of Soochow University, Suzhou, China; ^2^Department of Neurology, Suzhou Ninth People’s Hospital, Suzhou, China; ^3^Department of Neurology, The Affiliated Changzhou NO.2 People’s Hospital of Nanjing Medical University, Changzhou, China; ^4^Department of Neurology, Suzhou Guangci Cancer Hospital, Suzhou, China; ^5^Department of Neurology, The Dushu Lake Hospital of Soochow University, Suzhou, China; ^6^Department of Neurology, The Affiliated Taizhou Second People’s Hospital of Yangzhou University, Yangzhou, China

**Keywords:** lipid-lowering medications, epilepsy, drug target Mendelian randomization, causal, genetic, lipid metabolism

## Abstract

**Background:**

Previous research has yielded conflicting results on the link between epilepsy risk and lipid-lowering medications. The aim of this study is to determine whether the risk of epilepsy outcomes is causally related to lipid-lowering medications predicted by genetics.

**Methods:**

We used genetic instruments as proxies to the exposure of lipid-lowering drugs, employing variants within or near genes targeted by these drugs and associated with low-density lipoprotein cholesterol (LDL cholesterol) from a genome-wide association study. These variants served as controlling factors. Through drug target Mendelian randomization, we systematically assessed the impact of lipid-lowering medications, including HMG-CoA reductase (HMGCR) inhibitors, proprotein convertase subtilisin/kexin type 9 (PCSK9) inhibitors, and Niemann-Pick C1-like 1 (NPC1L1) inhibitors, on epilepsy.

**Results:**

The analysis demonstrated that a higher expression of HMGCR was associated with an elevated risk of various types of epilepsy, including all types (OR = 1.17, 95% CI:1.03 to 1.32, *p* = 0.01), focal epilepsy (OR = 1.24, 95% CI:1.08 to 1.43, *p* = 0.003), and focal epilepsy documented with lesions other than hippocampal sclerosis (OR = 1.05, 95% CI: 1.01 to 1.10, *p* = 0.02). The risk of juvenile absence epilepsy (JAE) was also associated with higher expression of PCSK9 (OR = 1.06, 95% CI: 1.02 to 1.09, *p* = 0.002). For other relationships, there was no reliable supporting data available.

**Conclusion:**

The drug target MR investigation suggests a possible link between reduced epilepsy vulnerability and HMGCR and PCSK9 inhibition.

## Introduction

Approximately 70 million people worldwide suffer from epilepsy, which has an annual rate ranging from 50.4 to 81.7 per 100,000 people (1, 2). Despite ongoing research, the precise mechanisms underlying epileptic seizures remain incompletely understood. Genetic predispositions, cerebrovascular illness, head traumas, and neurodegenerative disorders are only a few of the recognized causes of epilepsy. According to earlier research, metabolic variables may be very important in the development of epilepsy (3, 4).

Numerous observational studies have to date shown a link between higher circulating lipid levels and an enhanced risk of epilepsy. They have also suggested that the use of lipid-lowering medications might potentially alleviate seizure activity (5–13). Contradictory studies, however, have hypothesized that people with epilepsy could, in general, have lower amounts of circulating blood lipids. Additionally, there are evidence to suggest that increasing circulating blood lipids through the adoption of a ketogenic diet may result in a reduction in seizure frequency (14–16).

HMG-CoA reductase (HMGCR) inhibitors, sometimes referred to as statins, are a very frequently prescribed family of lipid-lowering medications. They offer various advantages, including well-established safety records, cost-effectiveness, and pleiotropic effects. Additionally, the proteins Niemann-Pick C1-like 1 (NPC1L1) and proprotein convertase subtilisin/kexin type 9 (PCSK9) play critical roles in controlling levels of circulating low-density lipoprotein cholesterol (LDL-C) (17, 18).

Genetic epidemiology provides an additional approach to address these questions. Variants located within or near genes responsible for encoding protein drug targets can potentially influence their expression or function. Genetic effects can serve as predictors of drug treatment outcomes (19). In contrast to other kinds of observational epidemiology, genotypes are randomly inherited at conception, much like the treatment allocation in clinical trials; thus, relationships between variations, biomarkers, and illness outcomes are less susceptible to confounding and reverse causation. The methodology known as “Mendelian randomization” (MR) is based on this premise (20). In addition, the general expectation is that genotypes contribute to the variation in traits throughout life. Consequently, by projecting the effects of prolonged therapeutic treatment, MR studies have the potential to inform the validation of pharmacological targets (21). The targets of drugs administered for primary hypercholesterolemia or familial hypercholesterolemia were studied in this study in relation to genetic variations. We also evaluated how they could affect epilepsy risk.

## Materials and techniques

### Research plan

**Figure 1:**
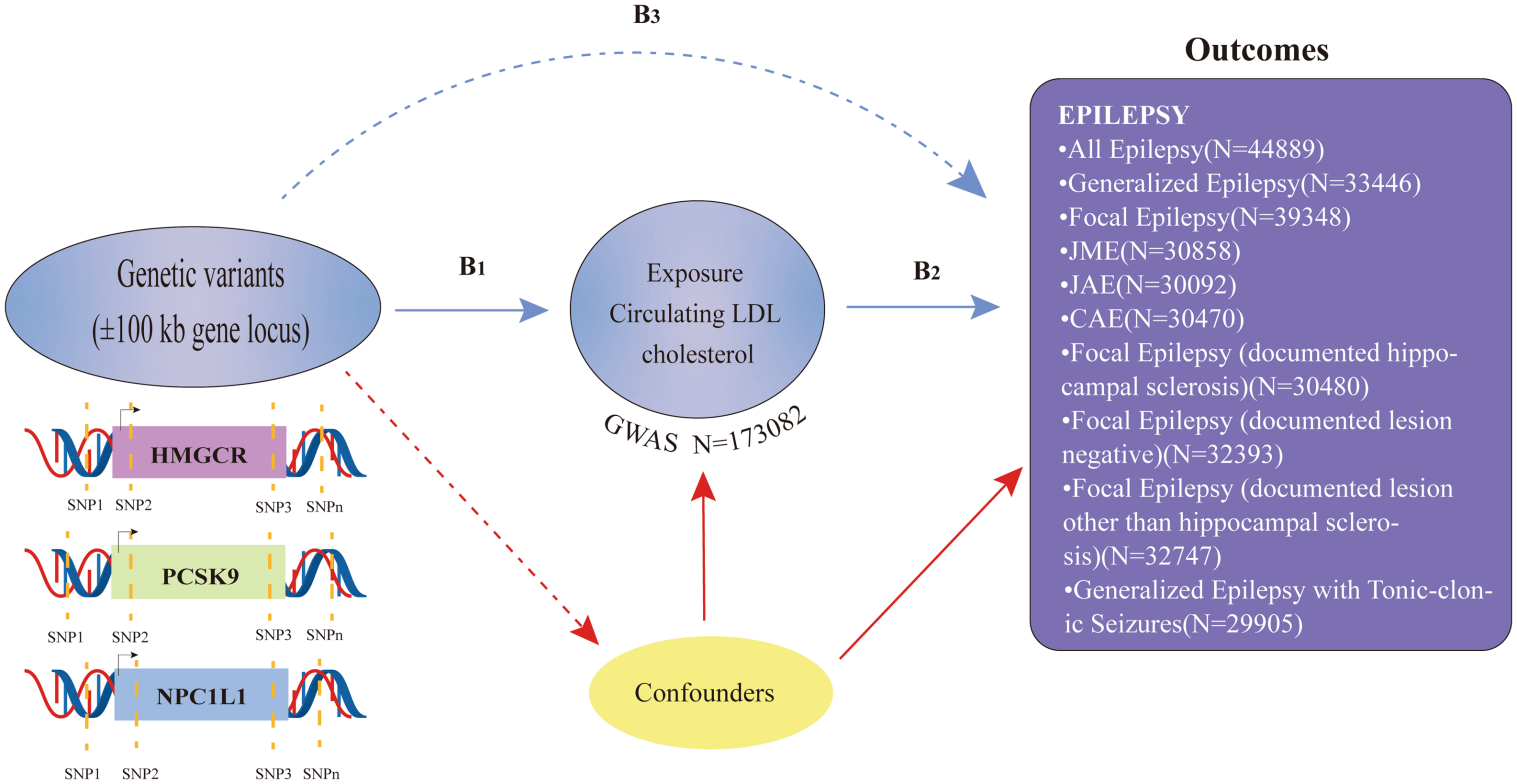
Study overview and Mendelian Randomization model.

In this two-sample MR analysis, publicly accessible summary-level data from genome-wide association studies (GWASs) were employed (refer to Supplementary Table S1). The following three guiding hypotheses served as the foundation for the MR analysis in this study: (i) the selected genetic variants should demonstrate a significant association with the exposure factor (LDL-C); (ii) the chosen genetic variants exhibit no association with other potential confounding factors.; and (iii) the chosen genetic variations only have an impact on the exposure component (LDL-C), and they have no additional effects on the outcome (epilepsy) (Figure 1). Only publicly available GWAS data were used in this study, and ethical approval and consent to participate could be found in the original GWAS study. The research was performed according to STROBE-MR guidelines (22).

### Genetic tool selection

Three kinds of lipid-lowering medications that have received FDA approval were used in this investigation as exposures. HMGCR inhibitors, PCSK9 inhibitors, and NPC1L1 inhibitors are the members of this class.

Our instrument selection procedure chose single-nucleotide polymorphisms (SNPs) located within 100 kb of the gene targeted by each medication, as shown in Supplementary Table S2. These selected SNPs demonstrated a genome-wide association with LDL-C levels (*p* < 5.0 × 10–8), making them suitable proxies when exposed to lipid-lowering drugs. The analysis relied on GWAS data for LDL-C levels with a sample size of 173,082 sourced from the Global Lipids Genetics Consortium (GLGC) for the identification of these SNPs. The research only took into account frequent SNPs with a minor allele frequency (MAF) higher than 1% (refer to Supplementary Table S2) (23).

Seven SNPs located within 100 kb of the HMGCR gene were chosen to serve as proxies for HMGCR inhibitors. There are 12 SNPs in the PCSK9 gene that have been shown to inhibit PCSK9. Additionally, to represent NPC1L1 inhibitors, three SNPs were selected from the NPC1L1 gene. To enhance the reliability of each instrumental variable for the respective medication, low linkage disequilibrium (*R*^2^ < 0.30) between the chosen SNPs was mandated.

### Sources of results

We compiled pooled statistics from the GWAS encompassing various forms of epilepsy. These data were sourced from the International League Against Epilepsy (ILAE) Consortium cohort (refer to Supplementary Table S1) (24, 25).

### Statistical analyses

#### Primary MR analysis

To determine causal estimates for the primary analysis, we used the inverse variance weighted (IVW) approach to random effects. Given that inhibitors targeting HMGCR, PCSK9, and NC1L1 are commonly used in the treatment of coronary artery disease (CAD), we utilized data from the CARDIoGRAMplusC4D CAD study, which encompasses 60,801 cases and 123,504 control subjects, primarily of European descent. This sample size of 60,801 cases served as a positive control group to assess the validity of the instruments related to HMGCR, PCSK9, and NC1L1 (26, 27). R-version 4.3.1 package was used for the analysis and assessment, and the MR and its MR packages were implemented (28, 29).

#### Analysis of sensitivity

Using the F-statistic, we evaluated the potency of SNPs used as instruments. To reduce weak instrumental bias, we took care to only include those with an F-statistic greater than 10 (30). The Cochran Q-test was used to assess the heterogeneity of certain SNPs. Potential pleiotropy was suggested by a Cochran Q-derived *p*-value of 0.05. It is important to note that pleiotropy does not introduce bias to the IVW’s estimates, as long as it remains balanced. We estimated the intercept using MR-Egger regression as a measure of directional pleiotropy to see if the pleiotropy was truly unbalanced. Statistical significance was defined as a value of p of 0.05 (31). If a notable outcome was observed, it would suggest the presence of imbalanced pleiotropy, which prompted us to conduct various sensitivity analyses to validate the findings derived from the IVW estimates. We used a variety of MR methods recognized for their resistance to pleiotropy in many sensitivity studies to confirm the validity of our findings, and Weighted Median Regression was one among the MR methods. It mandates that at least 50% of the weight used in the MR analysis come from reliable devices (32). Additionally, we employed MR-Egger regression, which is capable of detecting and adjusting for directional pleiotropy (33).

## Results

We retrieved a total of 7, 12, and 3 SNPs from the GWAS summary data of LDL-C levels in the GLGC. These SNPs were found within, close to, or near the HMGCR, PCSK9, and NPC1L1 genes, respectively (refer to Supplementary Table S2). All instrumental variations had F-statistics that were more than 30. This suggests that our study’s efforts to minimize possible weak instrument bias were successful (refer to Supplementary Table S2). The positive control study furnished compelling evidence linking drug exposure to coronary heart disease, utilizing instruments derived from LDL-C GWAS (refer to Supplementary Table S5). This evidence further substantiates the soundness of our chosen genetic instruments.

### Primary analysis

We started by lining up all of the outcome datasets with the exposures of the drug target instruments. We performed associated IVW-MR, MR-Egger, and Weighted Median MR experiments in the sections that follow. This process yielded test statistics for heterogeneity and pleiotropy. The standardized correlated MR effect estimates are presented last. According to these estimations, the medication target gene is indirectly inhibited by a one standard deviation (SD) decrease in biomarker LDL-C levels (SD = 38.7 mg/dL).

**Figure 2:**
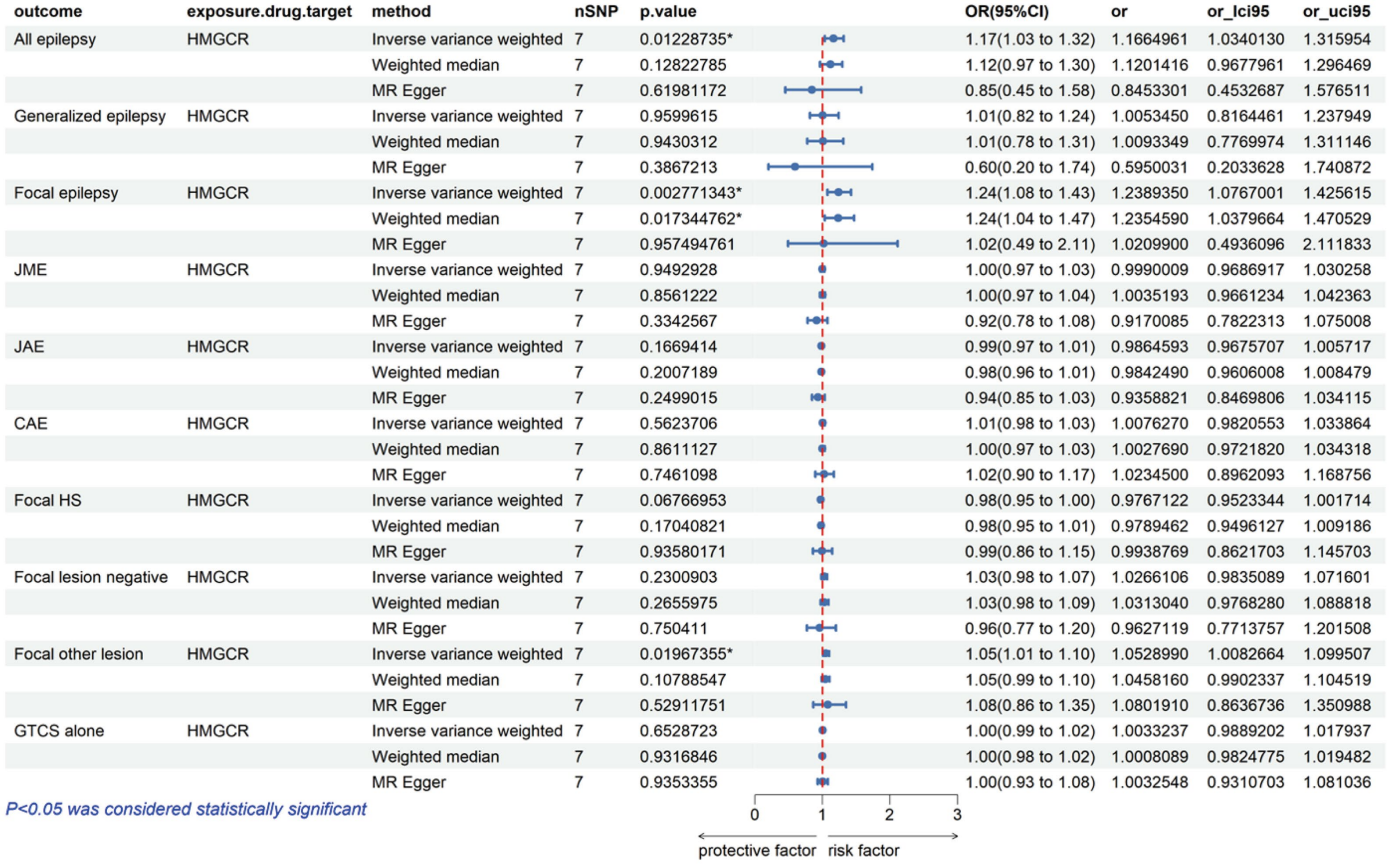
Potential impact of HMGCR inhibition on epilepsy risk.

**Figure 3:**
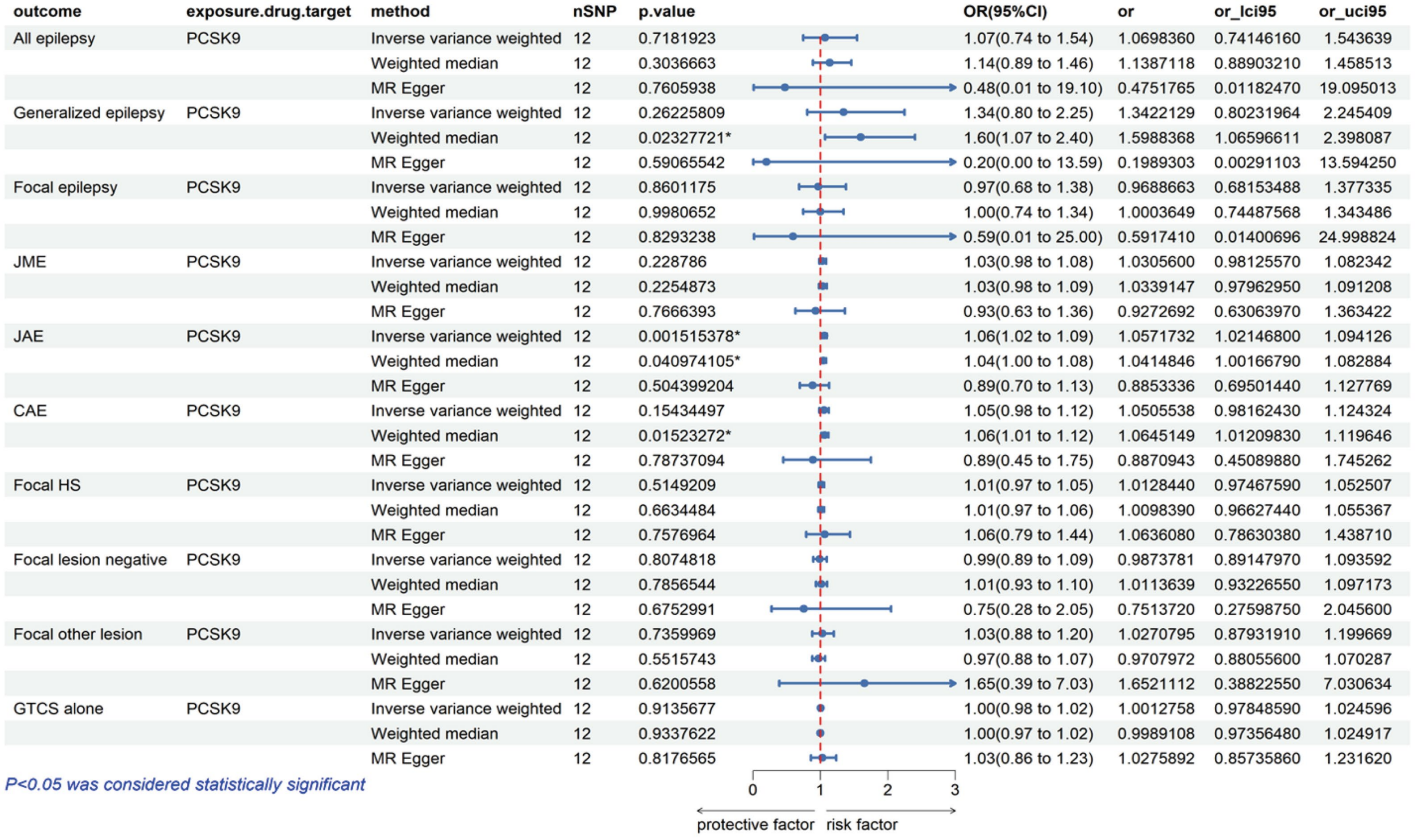
Potential impact of PCSK9 inhibition on epilepsy risk.

The analysis reveals that a higher expression of HMGCR is associated with an increased risk of various types of epilepsy, including all types, with an odds ratio (OR) of 1.17 with a 95% confidence interval (CI) of 1.03–1.32, accompanied by a corresponding *p*-value of 0.01. For focal epilepsy, the OR is 1.24 (95% CI = 1.08–1.43, *p* = 0.003), and for focal epilepsy with documented lesions other than hippocampal sclerosis, the OR is 1.05 (95% CI = 1.01–1.10, *p* = 0.02) (Figure 2; Supplementary Table S3). Similarly, as indicated in Figure 3 and Supplementary Table S3, for a heightened expression of PCSK9 that was linked with an elevated risk of juvenile absence epilepsy (JAE), the OR is 1.06 (95% CI = 1.02–1.09, *p* = 0.002). There was no evidence of an association between NPC1L1-mediated LDL-C and the outcome of epilepsy in Supplementary Figure S1 and Supplementary Table S3.

### Sensitivity assessment

The results consistently aligned across all applied Mendelian Randomization (MR) methods. The results of the Cochran Q-test did not indicate any evidence of heterogeneity in the epilepsy-related endpoints or the HMGCR-mediated LDL-C outcomes. All of the value of ps (refer to Supplementary Table S4) were higher than 0.05. However, some degree of heterogeneity emerged in the results concerning PCSK9-mediated LDL-C, encompassing both all types and focal epilepsy (excluding hippocampal sclerosis lesions) (Supplementary Table S4). Additionally, in the MR-Egger regression analysis, neither intercept term provided substantial evidence of pervasive horizontal pleiotropy (every value of p was higher than 0.05; refer to Supplementary Table S4).

## Discussion

Our drug target MR studies have identified a direct correlation between the risk of epilepsy and HMGCR, encompassing all types (OR = 1.17, 95% CI = 1.03–1.32, *p* = 0.01), focal epilepsy (OR = 1.24, 95% CI = 1.08–1.43, *p* = 0.003), and focal epilepsy characterized by documented lesions other than hippocampal sclerosis (OR = 1.05, 95% CI = 1.01–1.10, *p* = 0.02). These results indicate that HMGCR inhibitors might lower the risk of epilepsy. Moreover, our study has provided initial evidence supporting a positive correlation between PCSK9 expression and susceptibility to JAE (OR = 1.06, 95% CI = 1.02–1.09, *p* = 0.002). This result also suggests a potential risk reduction in epilepsy with the use of PCSK9 inhibitors. It reinforces the notion that these inhibitors could be a valuable consideration in epilepsy prevention. Conversely, we found no association between NPC1L1 expression and epilepsy outcomes.

Epilepsy, with its significant morbidity and mortality, is a prevalent chronic neurological condition. The ILAE describes it as a disorder brought on by either the breakdown of systems that should end seizures or the onset of mechanisms that should end excessively protracted seizures (34). With an estimated yearly prevalence of 800,000 occurrences of vascular epilepsy globally, stroke is also the main cause of acquired epilepsy in elderly people (35, 36). Statins, which are HMGCR inhibitors, have been commonly used for several years in the prevention of both ischemic and hemorrhagic strokes, according to randomized controlled studies and meta-analyses (37, 38). Furthermore, numerous studies have indicated enhanced neurological outcomes and a better acute stroke prognosis with statin administration (39, 40). Since neuroprotective agents have the capacity to influence the onset of epilepsy (41), statins have also been proposed for their potential anticonvulsant properties in epilepsy (42–46). Numerous neurosynaptic transmissions and molecular processes contribute to the antiepileptic properties of statins. PCSK9 inhibitors, serving as a crucial regulator of LDL-C, have become a significant focus in the development of cholesterol-lowering medications (17, 47, 48). Our MR study revealed a previously unreported protective role of PCSK9 inhibitors against JAE. Our study has identified a potential benefit of statins in reducing the risk of epilepsy. This finding suggests that statins could be considered a therapeutic option for patients with post-stroke epilepsy, particularly elderly patients. Additionally, it introduces a novel avenue for drug therapy in both clinical and preclinical studies.

As a genetic epidemiological method, MR overcomes the limitations of conventional observational studies. Previous clinical studies in epilepsy have suffered from varying designs, inconsistent terminology, limited sample sizes, fluctuating durations of follow-up, and uncertainty in the identification and classification of seizures, which has led to inconsistent results. Additionally, there is a limited amount of data available regarding particular subtypes of seizures. Our study effectively circumvents these issues. Previous attempts to establish a causal link between lipids and seizures using two-sample Mendelian randomization techniques yielded conflicting results (49). In this specific investigation, we utilized genetic variants associated with HMGCR-mediated LDL-C levels as instrumental proxies for statin exposure, effectively reducing the potential for confounding bias. This approach harnesses the wealth of currently available genetic information on epilepsy. Furthermore, our study addresses concerns of reverse causality and residual confounding through meticulous MR analysis. We applied various methodologies to validate MR estimates, ensure the consistency of our estimates across different MR models, and check for any violations of MR assumptions.

Genomic analytic techniques such as MR offer early evidence of long-term LDL-C modulation by drug targets; given the recent development of PCSK9 inhibitors, long-term trial data are still lacking. The consistency of our results across different MR methods, accommodating varied assumptions about genetic pleiotropy, significantly strengthens the causal inference of our analysis. Our research indicates that statins demonstrate a risk reduction in the occurrence of various forms of epilepsy, including all types, focal epilepsy, and focal epilepsy with documented lesions other than hippocampal sclerosis. The association between PCSK9 inhibitors and JAE has not been previously documented in existing literature. This unexplored area will be the central focus of our upcoming research endeavors and investigations.

### Research limitations

Our research contains a number of limitations, primarily stemming from the inherent assumptions and constraints of MR. First, while MR is a powerful tool for identifying genetic associations, it cannot fully replace rigorous, long-term randomized controlled trials for potential future gene-editing therapeutics. MR’s conclusions are contingent on the quality and scope of the underlying data. Second, the available data on epilepsy outcomes are relatively small and may lack the power needed to detect potential relationships adequately. It is therefore imperative to replicate these biometric analyses in epilepsy with larger datasets as they become available. Third, it is important to note that statins and PCSK9 inhibitors have limited penetration across the blood–brain barrier. Additionally, their mechanisms of action within the brain might not be reflected in our findings, which may not encompass tissue-specific correlations of statin and PCSK9 expression that could be altered in diseased brain states. Fourth, we admit that, despite our thorough sensitivity analyses that were created to carefully examine the fundamental assumptions of our MR study, there is still a chance of confounding bias and/or horizontal pleiotropy. Finally, it is crucial to note that the majority of the populations of European heritage were included in the GWAS data we used for this study. Consequently, we advise caution when generalizing these findings to other populations.

## Conclusion

In this study, we furnish genetic evidence supporting a worldwide as well as a regional link between lipid-lowering drugs such as HMGCR and PCSK9 inhibitors and epilepsy. Grasping this correlation offers potential for pioneering epilepsy treatments. Our MR research establishes a link between the risk of epilepsy and lipid-lowering medications.

## Data availability statement

The original contributions presented in the study are included in the article/Supplementary material, further inquiries can be directed to the corresponding author.

## Ethics statement

Ethical review and approval was not required for the study on human participants in accordance with the local legislation and institutional requirements. Written informed consent from the patients/participants or patients/participants’ legal guardian/next of kin was not required to participate in this study in accordance with the national legislation and the institutional requirements.

## Author contributions

SH: Conceptualization, Data curation, Writing – original draft. YL: Formal analysis, Investigation, Methodology, Writing – original draft. YZ: Formal analysis, Project administration, Resources, Writing – original draft. YW: Methodology, Project administration, Software, Writing – original draft. YG: Methodology, Project administration, Software, Writing – original draft. RL: Methodology, Project administration, Writing – original draft. LY: Resources, Software, Validation, Writing – review & editing. XH: Validation, Writing – review & editing. QF: Supervision, Writing – review & editing.

## Figures and Tables

**Table 1:** 

CAD	Coronary artery disease
CAE	Childhood absence epilepsy
F=F	statistic
FDA	Food and Drug Administration
GWAS	Genome-wide association study
GLGC	The Global Lipids Genetics Consortium
GTCS	Generalized tonic–clonic seizures alone
HMGCR	3-Hydroxy-3-methylglutaryl coenzyme A reductase
ILAE	The International League Against Epilepsy
IVs	Instrumental variables
IVW	Inverse-variance weighted
JME	Juvenile myoclonic epilepsy
JAE	Juvenile absence epilepsy
LDL-C	Low-density lipoprotein cholesterol
MR	Mendelian randomization
MAF	Minor allele frequency
NPC1L1	Niemann–Pick C1-like 1
OR	Odds ratio
PCSK9	Proprotein convertase subtilisin/kexin type 9
R2	Percentage of the variation explained by the SNP
SNPs	Single-nucleotide polymorphisms
STROBE-MR	Strengthening the Reporting of Observational Studies in Epidemiology using Mendelian Randomization
