# Supplementary figures and images for: Analyzing the causal relationship between lipid-lowering drug target genes and epilepsy: a Mendelian randomization study
Source: Front Neurol. 2024 Mar 8;15:1331537. doi: 10.3389/fneur.2024.1331537 (PMC10957583; doi:10.3389/fneur.2024.1331537)

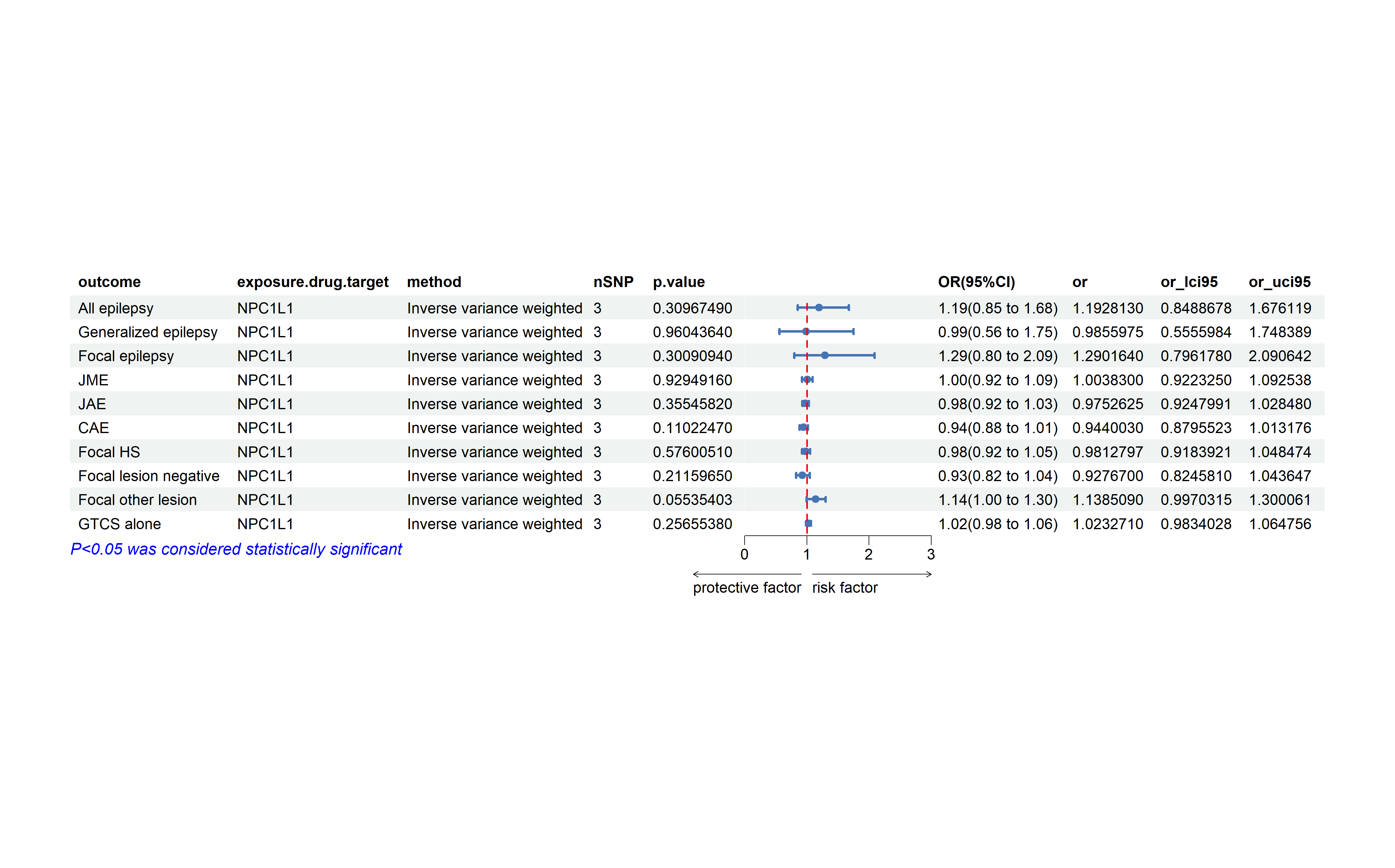

Supplement: Supplementary file 2 [file Image_1.tif]
